# Botulinum Toxin Treatment of Notalgia Paresthetica—A Critical Review and Update

**DOI:** 10.3390/toxins18010050

**Published:** 2026-01-19

**Authors:** Ava Grace Tohidian, Shahroo Etemadmoghadam, Bahman Jabbari

**Affiliations:** 1 Royal College of Surgeons, School of Medicine, D02 YN77 Dublin, Ireland; avagtohidian@gmail.com; 2 Glenn Biggs Institute, University of Texas Health San Antonio, San Antonio, TX 78229, USA; shahrooetemad@yahoo.com; 3 Department of Neurology, Yale University School of Medicine, New Haven, CT 06519, USA

**Keywords:** notalgia paresthetica, botulinum toxin, botulinum neurotoxin, onabotulinumtoxinA, incobotulinumtoxinA, pruritus

## Abstract

Notalgia paresthetica is a condition characterized by pruritus and pain in the upper back, often associated with skin discoloration in the same area. Through Medline, Google Scholar, and Scopus search engines, we identified reports of eight clinical studies (published up to 1 December 2025) on the subject of botulinum neurotoxin therapy for Notalgia Paresthetica (NP). Only one of the eight studies was double-blind and placebo-controlled. The search strategy included only articles published in English and Spanish, and articles providing basic information such as the type of study, type and dose of the toxin, and results of the treatment. Articles not in English or Spanish, review articles, and articles failing basic information were excluded. A total of 34 patients were found across all studies. The injected toxin in the open-label studies was onabotulinumtoxin-A (Botox), whereas in the blinded study, the investigators used incobotulinumtoxinA (Xeomin). All open-label studies reported improvement in pruritus, and some reported improvement in pain, whereas the blinded study failed to do so. The possible reasons for this discrepancy between the blinded and the open-label studies are discussed. There is a need for double-blind, placebo-controlled studies with a larger number of patients, preferably using the same neurotoxin that has suggested efficacy in the open-label studies. The novelty of this review is that it represents a comprehensive and critical literature assessment on this topic and that it includes data not present in the previous reviews of this subject.

## 1. Introduction

**Figure 1:**
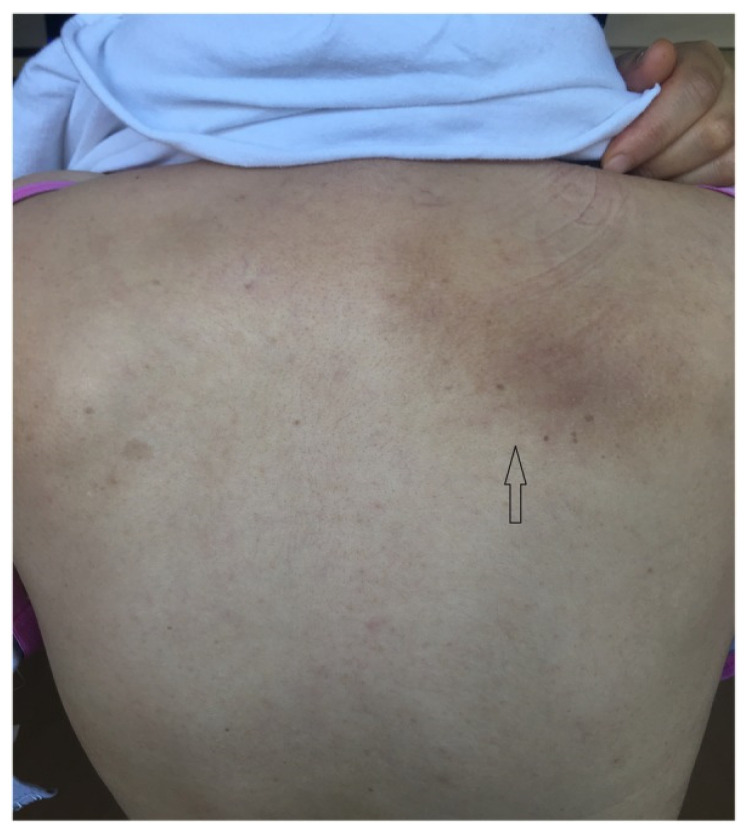
Pruritic, hyperpigmented skin (arrow) in a patient with Notalgia Paresthetica. From Mülkoğlu and Nacır, BMC Neurol 2020 [5]. Reproduced under the Creative Commons Attribution License 4.0 (http://creativecommons.org/licenses/by/4.0/, accessed on 4 January 2026)—Courtesy of the publisher (Springer Nature).

Notalgia paresthetica (NP) is a neuropathic condition characterized by pruritus primarily in the middle and upper areas of the back, between the shoulder blades. It may present with or without a patch of hyperpigmentation or lichenification of the skin, usually coinciding with the area of pruritus [1] (Figure 1). The prevalence of NP is not well established. The condition is more common among white women of middle age, with a mean age of onset between 55 and 60 years [2]. The clinical presentation primarily includes chronic, localized pruritus, often accompanied by localized burning, pain, tingling, or numbness. NP is chronic and often persistent, with symptoms that can last months to years [3,4].

The current first-line treatment is topical anesthetics (e.g., lidocaine), which can provide short-term relief [6]. Topical capsaicin, calcineurin inhibitors (e.g., tacrolimus), and oral neuromodulators (gabapentinoids) are also used; however, they have variable efficacy and unfavorable side effects, which lower adherence [5,7]. More invasive treatments are considered for refractory cases. These may include intradermal lidocaine, acupuncture, and neural therapy [8,9]. Non-pharmacological therapies such as physical therapy, muscle strengthening, and postural correction may also provide partial relief [10]. The effectiveness of the above-mentioned available treatments for notalgia paresthetica is limited, with most therapies providing only partial or temporary relief [11]. Since subcutaneous or intradermal injections of botulinum toxins have been shown to be capable of alleviating neuropathic itch and pain [11,12,13], their efficacy in relieving symptoms of NP has been the subject of investigation over the past two decades.

## 2. Research Design

We have searched Medline, Scopus, and Google Scholar up to 1 December 2025. The search terms consisted of botulinum toxin as well as botulinum neurotoxin, and notalgia paresthetica. Two of the authors independently searched the literature (AGT, BJ), while a third author (SE) verified the search results. Excluded from the search were articles in languages other than English and Spanish, and the reports that failed to mention toxin type and dose. The strengths and weaknesses of the searched studies, as well as the technical issues associated with BoNT therapy in NP, are provided in the discussion part of this manuscript. The novelty of this review is that it presents both a critical review and a literature update on the subject of NP.

## 3. Results

We found eight manuscripts that conformed to the research criteria. These articles were published between 2007 and 2025. Table 1 represents a summary of search results. It includes authors’ names and dates of publication, number of patients, study type, toxin type, total dose of the toxin, sites of injection, primary outcome, and results. There was one double-blind placebo-controlled study, three prospective studies, and four retrospective studies.

## 4. Discussion

A disturbing itch is the most prominent symptom of Notalgia Paresthetica (NP), which in most patients is felt below the scapula (Figure 1). It is currently believed that the type of itch in NP is neuropathic since the cause of NP is believed to be either pressure against T2–T6 nerve roots from neighboring spinal pathology or entrapment of the nerves originating from these roots inside a tight upper back muscle [22].

The sensation of neuropathic itch is conveyed to the spinothalamic tract and central nervous system via small unmyelinated histamine-sensitive C fibers [23]. Like neuropathic pain, recurrent sensation of itch leads to sensitization of the itch-perceiving neurons in the central nervous system (central sensitization); as a result, excessive response to normal or subthreshold stimuli promotes itch chronicity. In a study of 45 patients with NP and chronic pruritus, using a 25-item validated central sensitization inventory (CSI), the authors found that central sensitization (CS) was significantly higher in NP patients compared to controls (*p* = 0.037) [24]. Local injections of botulinum toxin relieve neuropathic pain [25,26], an effect attributed to reduced action of substance P and calcitonin gene-related peptide (CGRP), pain and itch neurotransmitters for C fibers. Furthermore, it has been shown that in both human and animal pain models, local injection of botulinum toxin A significantly reduces the phenomenon of central sensitization [27,28].

**Figure 2:**
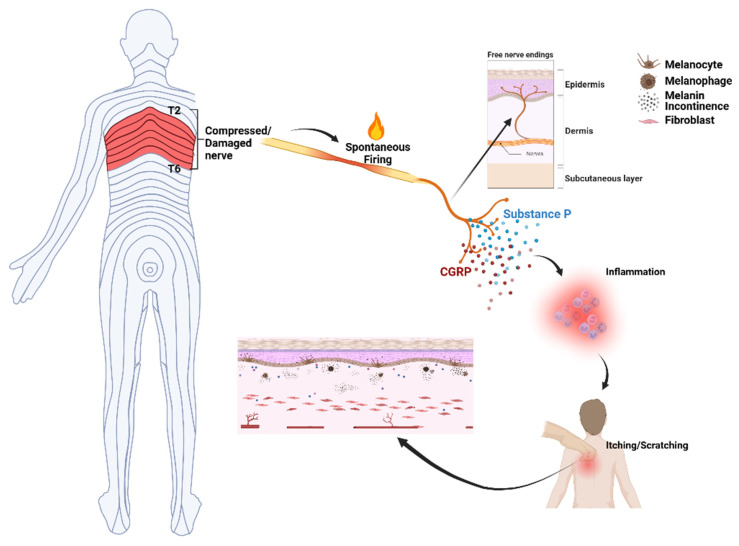
Hypothetical and schematic presentation of pathological findings in NP based on the information from recent publications [29,30,31]. Image created in BioRender. Etemad, S. (2026) https://BioRender.com/nzhfteh (accessed on 4 January 2026).

The literature on skin pathology in NP is limited. Reported findings consist of thinning of the epidermis, epidermal hyperpigmented keratocytes, pro-inflammatory melanosis, presence of dermal melanophages, as well as dermal amyloid deposits and decreased numbers of dermal nerve fibers in the pruritic area [29,30,31] (Figure 2).

Local injection of botulinum toxin A has been shown to reduce local inflammation in different animal models. In rats, local injection of onabotulinum toxin A, prior to formalin injection, reduced the local inflammatory response as well as local pain in the injected paw [32]. In adjuvant-induced arthritic pain, local injection of botulinum toxin A ameliorated microglial activation and local inflammation [33]. Local injection of botulinum toxins downregulates TRP1 and TRV1 transient receptor channels that are involved in the transmission of itch signals triggered by histamine itch via specific receptors (HIR and H4R) [34]. In rosacea, a chronic skin condition, local injection of botulinum A or B blocks mast cells and prevents the development of new skin lesions [35]. In psoriasis, a more common skin lesion than NP, several studies have reported improvement of psoriatic symptoms (including pruritus) and skin lesions following injection of different types of As and type B botulinum toxin into the skin [36]. In murine models of psoriasis, injection of either botulinum toxin A or B into psoriatic lesions reduces cytokine levels as well as local levels of CD4, CD11, and IL-17 in the skin [37,38].

Our search identified eight publications directly related to the topic of botulinum toxin therapy for NP. Seven observations, all open-label and conducted in a small number of patients, claim that botulinum toxin injection into the skin relieves patients’ pruritus (Table 1) up to 3 months or longer. Against these positive observations is the negative double-blind, placebo-controlled study of Maari et al. [17] (Table 1). The reason for the discrepancy between open-label observations and the blinded study is not clear. There are several possible explanations: 1—The information from open-label studies is subject to bias due to the possibility of the placebo effect (particularly relevant to pain and pruritus), heterogeneity of the group, and the fact that some studies did not use standard assessments. 2—The negative result of the blinded study [17] might have been caused by its low power due to the small number of studied patients in each studied group (nine toxin, nine saline). 3—The blinded study cited above used incobotulinumtoxinA (incoA), whereas the toxin used in the open-label observations was onabotulinumtoxinA (onaA). Although the doses of incoA and onaA are considered roughly comparable, in reality, the doses of different botulinum toxins are not interchangeable [39]. Furthermore, since success in BoNT therapy is dose-dependent, meaningful comparisons are not possible across studies as doses varied significantly, and in some studies, the total dose used was not even mentioned.

Over the past 10 years, treatment strategies for the management of NP have been published in several review articles [11,30,40,41,42,43,44]. Among different modes of treatment for NP, most of these reviews briefly included treatment with BoNTs [11,30,40,41,42,43,44], while two reviews addressed only BoNT therapy in NP [42,45]. Among the general reviews, three reviews did not mention BoNT treatment. The other four general reviews of NP briefly discussed three–five studies, with some lacking important information such as toxin type, treated area, duration of treatment, and adverse effects. Of the two reviews that focused specifically on BoNT therapy in NP, one briefly reviewed four studies, and the other briefly reviewed five studies. None of the studies discussed the issue of discrepancy between open-label investigations and the blinded study, or offered possible explanations. We believe the novelty of our review is in its more critical assessment of the data and in providing information missing in previous reviews.

Several studies have emphasized the positive effect of physiotherapy (PT) in NP. Zagarella et al. [45] inducted 12 patients with NP in a 12-week home physiotherapy program, comprising daily strengthening exercises for the upper back and shoulder muscles, as well as a weekly massage. At the end of the 12 weeks, 11 out of 12 patients reported a significant improvement in their symptoms. In another study [46], 65% of the patients with NP following 6 weeks of physiotherapy noted a satisfactory reduction in disturbing NP symptoms. The PT program included application of targeted pressure and manipulation of muscle spasms that enhanced the motility of the costovertebral joint and thoracic facets. Combining botulinum toxin therapy with targeted physiotherapy may enhance the effect of the former approach, which has not been adopted in any of the reported studies on botulinum toxin therapy in NP.

The weakness of our review is limiting the search to manuscripts written in English and Spanish, and the fact that most of the retrieved literature encompasses a small number of patients and unblinded observations that bias the findings. The strength of this review lies in its critical discussion of discordant findings and the inclusion of the most recent reports.

## 5. Conclusions

Although several open-label observations suggest that subcutaneous and intradermal BoNT injections can diminish the intensity of pruritus in NP, inherent problems with these studies, such as a placebo effect, lack of standard assessment methods, and heterogeneity of the studies, biased the conclusions. Blinded studies with a sizable number of patients are needed to discern the role of botulinum toxin therapy in Notalgia Paresthetica. It would be preferable to conduct such studies with onabotulinumtoxinA, the same toxin, which in the unblinded studies suggested a beneficial effect in the treatment of NP. Admittedly, studies with a large number of patients would be difficult to conduct in NP due to the uncommon nature of NP.

## Figures and Tables

**Table 1:** Summary of Articles Identified in the Literature that Meet the Inclusion and Exclusion Criteria.

Authors and Date	Study Type	No. of Pts	Toxin Type	Total Dose	Injected Site	Assessment Methods	Results
Weinfeld et al., 2007 [14]	Retro	2	BoNT-A	Pt 1: 16 U. Pt 2: first treatment, 24 U; second treatment, 48 U.	Intradermal injections, 2 cm apart in the affected pruritic area of the upper back.	Intensity of pruritus by VAS at baseline and 8, 12, 18, and 24 weeks post-injection.	Patient 1: Full remission a week after treatment. Patient 2: Partial remission after the first treatment, but full remission after the second treatment.
Wallengren et al., 2010 [15]	Pros	4	BoNT-A	18–100 U	Intradermal injection 0.8–1.4 units, 1–5 cm apart.	Intensity of itch using VAS at weeks 1 and 6 after treatment.	One patient was free of itching at 6 and 18 weeks post-injection. Three patients showed 28–45% improvement of VAS at 6 weeks.
Perez-Perez et al., 2013 [16]	Pros	5	Ona-A	Varied depending on the size of skin involvement.	Intradermal 2 cm apart; four units were injected per site.	Intensity of pruritus by VAS at baseline and 1, 6, 12, and 18 months.	Two patients: >80% reduction in VAS score at one month, 60–70% reduction at 3 months. Two other patients: 50% reduction in VAS score at 6 months (judged by the authors’ figure).
Maari et al., 2014 [17]	DB-PC; COD	18	incoA: 9 Saline: 9	Mean dose: 142 U (max 200 U).	Intradermal injections every 1–2 cm into the hyperpigmented or pruritic areas of the upper back (T2–T6 dermatomes).	Baseline VAS compared to VAS at 8, 12,16, and 24 months post-treatment.	No significant change with treatment.
Morcillo-Pérez et al. 2019 [18]	Pros	2	Ona-A	Total dose based on the extension of the lesion.	Intradermal injections of five units, 1 cm apart, into the hyperpigmented pruritic region.	Intensity of pruritus recorded as mild, moderate, and severe intensity of pain measured on a 0–10 scale.	Case 1: Pruritus changed from moderate to severe to absent; pain changed from grade 4 to absent both at 3 and 6 months post-injection. Case 2: Pruritus changed from moderate to absent, and pain changed from 5 to absent at 3 and 6 months post-injection.
Datta et al., 2020 [19]	Retro	1	Ona-A	Not mentioned.	Intradermal injections, 1–2 cm apart; each site received two–five units.	Intensity of pruritus.	Pruritus significantly improved.
Sharad, J, 2020 [20]	Retro	1	Ona-A	100 U	Intradermal injections, 1 cm apart, into the lesion area (3 × 5 cm).	Intensity of pruritus.	Pruritus significantly improved.
Moreno et al., 2025 [21]	Retro	1	Ona-A	First session: 10 U. Second session two weeks later: 18 U.	Intramuscular (right upper back).	Intensity and frequency of pruritus on a scale of 1 to 10.	Initial pruritus intensity: 8 out of 10. Three weeks post-injection: 1–2 out of 10.

DB-PC: double-blind, placebo-controlled; COD: crossover design; Retro: retrospective, Pro: prospective; ona: onabotulinumtoxinA (Botox); incoA: incobotulinumtoxinA (Xeomin); BoNT-A: Botulinum neurotoxin A (type not specified).

## Data Availability

No new data were created or analyzed in this study.
